# Supplementary material for: The Potyviruses: An Evolutionary Synthesis Is Emerging
Source: Viruses. 2020 Jan 22;12(2):132. doi: 10.3390/v12020132 (PMC7077269; doi:10.3390/v12020132)
Supplement: Supplementary file 1 [file viruses-12-00132-s001.zip › viruses-696740-suppl/viruses-696740.Figure S1.pdf]

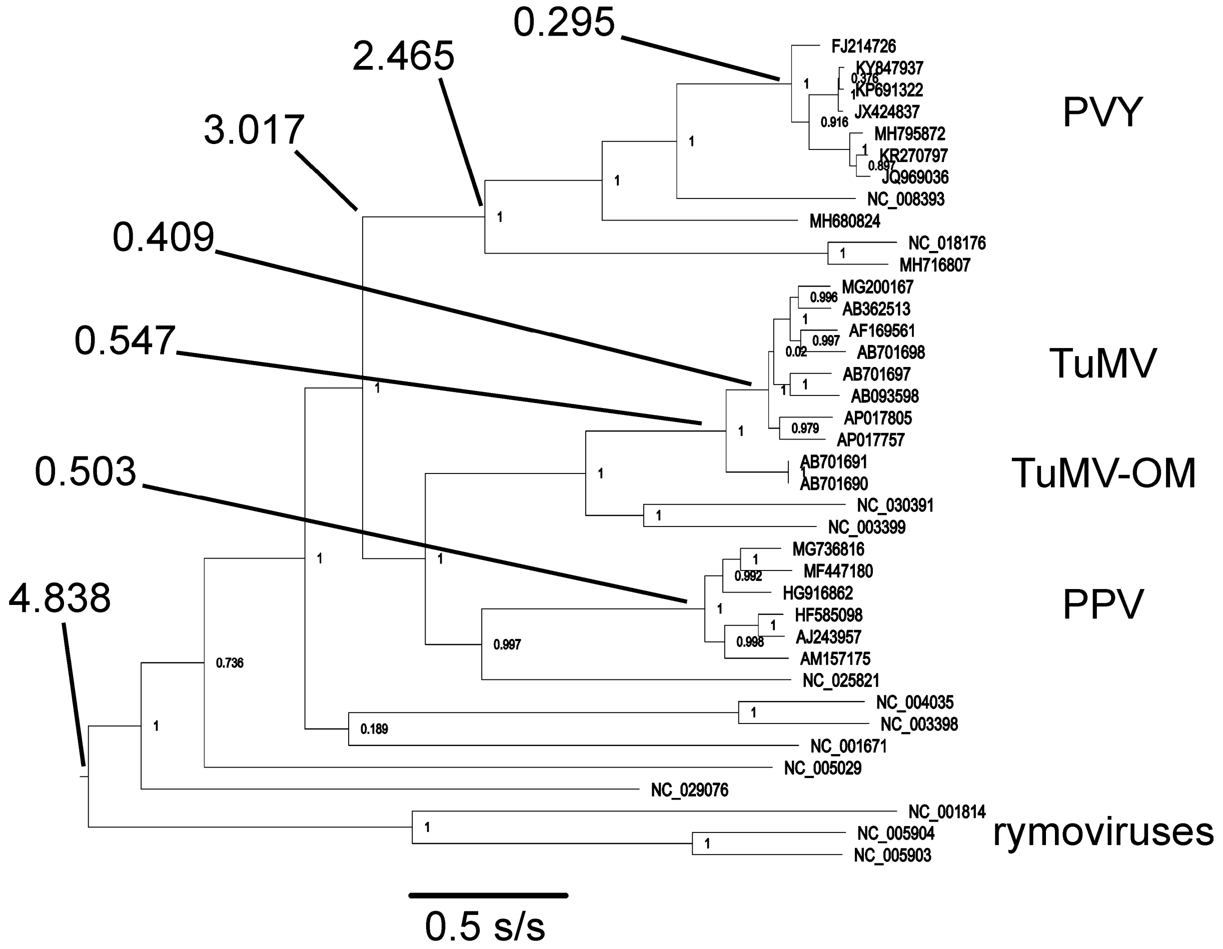

Fig S1 Subtree comparisons.

A ML tree of 38 potyvirus ORFs representative of the phylogroups of PVY, the groups of TuMV, the strains of PPV and outgroups. The basal nodes of each virus are marked with the mean pairwise patristic distance of all terminal leaves connected through that node. The immediate outgroup of the seven PVY sequences (patdist 0.295s/s) are four sequences from the PVY lineage of American viruses with the arracacha viruses being sister to all the others (patdist 2.465s/s). All other viruses (TuMV, PPV, etc plus rymoviruses) are probably Eurasian in origin, thus the migration of the PVY lineage progenitor from Eurasia to the Americas occurred between basal node of the PVY lineage (2.465s/s) and the nearest Eurasian node (3.017s/s). The midpoint (4.838s/s) of the entire tree represents the divergence of the potyviruses and their sister genus the rymoviruses.

The date of one node can be extrapolated to others by comparing patristic distances, so that if the TMRCA of PVY is taken as 156 CE, namely 1864 YBP, then protoPVY migrated to the Americas between 15.6 kYBP and 19.1 kYBP and the potyviruses and rymoviruses diverged 30.6 kYBP. If the TuMV TMRCA of 1201 YBP is used then the migration to the Americas occurred between 7.7 kYBP and 8.8 kYBP and the poty:rymo split was at 14.2 kYBP.

AB093598 TuMV Al
AB362513 TuMV TUR9
AB701690 TuMV-OM
AB701691 TuMV-OMA
AB701697 TuMV ASP
AB701698 TuMV BEL1
AF169561 TuMV UK1
AJ243957 PPV M-PS
AM157175 PPV El Amar
AP017757 TuMV IRNBRE4
AP017805 TuMV IRNTSh8
FJ214726 PVY Chile 3
HF585098 PPV Dideron
HG916862 PPV 1410-7
JQ969036 PVY 10N
JX424837 PVY O UK
KP691322 PVY CRM2
KR270797 PVY mar7
KY847937 PVY CO11
MF447180 PPV Tat-4
MG200167 TuMV KBJ2
MG736816 PPV Pul-DS
MH680824 MashuaVY Cam
MH716807 ArraVY 19384
MH795872 PVY Czo24
NC_001671 PSbMV
NC_001814 RGMV
NC_003398 SCMV
NC_003399 ScMV
NC_004035 SgMV
NC_005029 OYDV
NC_005903 AgMV
NC_005904 HordMV
NC_008393 PepSMV
NC_018176 ArraMoV
NC_025821 AspV1
NC_029076 ISMV
NC_030391 WOSV
