# Supplementary material for: The Potyviruses: An Evolutionary Synthesis Is Emerging
Source: Viruses. 2020 Jan 22;12(2):132. doi: 10.3390/v12020132 (PMC7077269; doi:10.3390/v12020132)
Supplement: Supplementary file 1 [file viruses-12-00132-s001.zip › viruses-696740-suppl/viruses-696740.Table S1-4.pdf]

Supplementary Data

Tables S1-4

Results of population genetics analyses of PVY, TuMV, SwPVs and HarMV

S1.1 Genetic diversity of PVY populations based on the polyprotein

Phylogroups	N	H	Hd	S	η	K	π	SS	NS	d_S	d_N	d_N/d_S	Tajima's D[a]
**Os**	136	134	0.999	2727	3028	249.43	0.027	2063.72	7119.28	0.098	0.007	0.068	-1.823*
**Ns**	26	26	1.000	1346	1403	379.04	0.043	1983.13	6929.87	0.165	0.008	0.48	0.124[ns]
**Cs**	14	14	1.000	3294	3865	1034.11	0.113	2058.26	7112.74	0.391	0.032	0.082	-0.677ns
**Os+Ns+Cs**	162	160	0.999	3190	3789	566.56	0.064	1994.49	6918.51	0.232	0.015	0.065	-0.503ns
**R1**	105	104	0.999	2216	2391	178.88	0.019	2054.96	7074.05	0.063	0.007	0.109	-2.069*
**R2**	119	116	0.999	2105	2256	151.10	0.017	2011.14	6961.86	0.056	0.006	0.101	-2.157**
**R1+R2**	224	220	0.999	3127	3518	379.61	0.042	2012.49	6957.51	0.152	0.011	0.070	-1.135[ns]

[a]ns: Not significant

N: Number of sequences, H: Number of haplotypes, Hd: Haplotype diversity, S: Number of polymorphic (Segregating) sites, η: Number of mutations, K: Average number of nucleotide differences between sequences, π: Nucleotide diversity, SS: Number of synonymous sites, NS: Number of non-synonymous sites, d_N: Non-synonymous nucleotide diversity, d_S: Synonymous nucleotide diversity

S1.2 Genetic diversity and genetic distances within and between the PVY populations

	Within PVY populations	Between PVY populations				
		**Os**	**Ns**	**Cs**	**R1**	**R2**
**Os**	0.0272 (0.000)	…	0.003	0.002	0.002	0.003
**Ns**	0.0433 (0.002)	0.165	…	0.003	0.003	0.002
**Cs**	0.0113 (0.000)	0.123	0.173	…	0.002	0.003
**R1**	0.0196 (0.001)	0.067	0.127	0.142	…	0.002
**R2**	0.00177 (0.000)	0.114	0.084	0.154	0.067	…

S1.3 Genetic differentiation measurement for PVY populations

Phylogroups	[a]Ks* (*P*-value)	[a]Z* (*P*-value)	[a]Snn (*P*-value)	[b]Fst
Os (n= 136) vs. Ns (n= 26)	5.146 (0.000***)	8.174 (0.000***)	1.000 (0.000***)	0.786
Os (n= 136) vs. Cs (n= 14)	5.867 (0.000***)	8.212 (0.000***)	0.993 (0.000***)	0.435
Os (n= 136) vs. R1 (n= 105)	5.042 (0.000***)	8.816 (0.000***)	1.000 (0.000***)	0.651
Os (n= 136) vs. R2 (n= 119)	4.956 (0.000***)	8.688 (0.000***)	1.000 (0.000***)	0.806
Ns (n= 26) vs. Cs (n= 14)	5.912 (0.000***)	5.158 (0.000***)	1.000 (0.000***)	0.548
Ns (n= 26) vs. R1 (n= 105)	4.810 (0.000***)	7.704 (0.000***)	1.000 (0.000***)	0.769
Ns (n= 26) vs. R2 (n= 119)	4.794 (0.000***)	7.940 (0.000***)	1.000 (0.000***)	0.657
Cs (n= 14) vs. R1 (n= 105)	5.117 (0.000***)	7.715 (0.000***)	1.000 (0.000***)	0.533
Cs (n= 14) vs. R2 (n= 119)	4.963 (0.000***)	7.953 (0.000***)	1.000 (0.000***)	0.577
R1 (n= 105) vs. R2 (n= 119)	4.796 (0.000***)	8.432 (0.000***)	1.000 (0.000***)	0.739

[ns], not significant; *, 0.01<P<0.05; **, 0.001<P<0.01; ***, P<0.001; [a]K*, Z*, and Snn are test statistics of genetic differentiation; [b]F_{ST}, coefficient of gene differentiation, which measures inter-population diversity

S1.4 Genetic diversity of PVY populations based on each gene separately

- P1 (825 nt)

Phylogroups	N	H	Hd	S	η	K	Π	SS	NS	d_S	d_N	d_N/d_S	Tajima's D[a]
**Os**	136	108	0.992	370	431	34.27	0.042	198.67	626.33	0.105	0.022	0.150	-1.860*
**Ns**	23	20	0.988	223	233	47.21	0.057	196.60	628.40	0.140	0.031	0.222	-1.022ns
**Cs**	14	14	1.000	449	568	139.11	0.170	193.01	622.99	0.404	0.098	0.242	-1.001ns
**Os+Ns+Cs**	173	142	0.995	554	814	94.65	0.116	196.70	619.30	0.274	0.066	0.241	-1.086ns
**R1**	105	84	0.984	311	342	56.59	0.068	197.71	627.29	0.148	0.044	0.295	-0.455ns
**R2**	119	92	0.988	245	272	30.68	0.043	198.12	626.88	0.109	0.031	0.281	-1.315ns
**R1+R2**	222	178	0.994	393	456	52.69	0.064	197.92	627.08	0.139	0.040	0.289	-0.983ns

- HC-Pro (1395 nt)

Phylogroups	N	H	Hd	S	η	K	Π	SS	NS	d_S	d_N	d_N/d_S	Tajima's D[a]
**Os**	136	119	0.995	424	465	33.81	0.024	301.65	1093.35	0.097	0.004	0.044	-1.984*
**Ns**	26	23	0.988	212	219	60.62	0.043	304.46	1090.55	0.173	0.007	0.042	0.223ns

**Cs**	14	14	1.000	508	582	163.45	0.117	301.90	1093.10	0.444	0.027	0.060	-0.484ns
**Os+Ns+Cs**	176	156	0.996	611	779	103.12	0.074	302.09	1092.91	0.279	0.017	0.060	-0.777ns
**R1**	105	96	0.995	355	385	20.04	0.014	303.65	1091.35	0.053	0.003	0.067	-2.453**
**R2**	119	106	0.998	310	334	14.51	0.011	292.80	1051.20	0.042	0.002	0.049	-2.554***
**R1+R2**	224	200	0.998	486	547	17.82	0.013	292.66	1051.34	0.050	0.003	0.056	-2.562***

- P3 (1095 nt)

Phylogroups	N	H	Hd	S	η	K	Π	SS	NS	d_S	d_N	d_N/d_S	Tajima's D[a]
**Os**	136	107	0.989	289	309	23.57	0.022	257.15	837.85	0.074	0.005	0.073	-1.911*
**Ns**	26	20	0.972	165	172	49.97	0.046	251.08	843.92	0.168	0.009	0.054	0.429ns
**Cs**	14	14	1.000	415	484	123.16	0.113	252.01	839.99	0.342	0.044	0.129	-0.863ns
**Os+Ns+Cs**	176	141	0.993	500	649	70.41	0.064	254.92	837.08	0.216	0.018	0.086	-1.222ns
**R1**	105	83	0.978	224	239	18.33	0.017	257.89	837.11	0.057	0.004	0.074	-2.006*
**R2**	119	97	0.991	256	271	16.86	0.015	257.82	837.18	0.049	0.005	0.099	-2.213**
**R1+R2**	224	179	0.993	367	406	19.01	0.017	257.85	837.15	0.057	0.005	0.089	-2.284**

- PIPO (231 nt)

Phylogroups	N	H	Hd	S	η	K	Π	SS	NS	d_S	d_N	d_N/d_S	Tajima's D[a]
**Os**	136	49	0.904	58	60	5.27	0.023	52.46	175.54	0.026	0.022	0.829	-1.915ns
**Ns**	26	9	0.865	27	28	9.07	0.039	52.93	178.07	0.053	0.035	0.656	0.087ns
**Cs**	14	14	1.000	99	116	26.06	0.113	52.69	175.31	0.155	0.099	0.636	-1.276ns
**Os+Ns+Cs**	176	72	0.940	117	148	14.65	0.064	52.50	175.50	0.087	0.055	0.638	-1.367ns
**R1**	105	37	0.794	33	35	2.53	0.011	52.97	175.03	0.009	0.011	1.207	-1.911*
**R2**	119	43	0.786	40	42	2.20	0.009	52.73	175.27	0.008	0.009	1.072	-2.216**
**R1+R2**	224	71	0.801	59	63	2.41	0.010	52.84	175.16	0.009	0.011	1.078	-2.309**

- CI (1902 nt)

Phylogroups	N	H	Hd	S	η	K	Π	SS	NS	d_S	d_N	d_N/d_S	Tajima's D[a]
**Os**	136	124	0,997	541	600	50.18	0.026	434.25	1467.76	0.106	0.003	0.025	-1.790*
**Ns**	26	23	0.991	293	304	84.44	0.044	432.62	1469.38	0.179	0.005	0.025	0.238ns

**Cs**	14	14	1.000	622	731	206.39	0.108	435.42	1466.58	0.415	0.017	0.042	-0.462ns
**Os+Ns+Cs**	176	161	0.998	770	1006	136.52	0.072	434.10	1467.90	0.281	0.009	0.035	-0.716ns
**R1**	105	90	0.991	365	377	23.60	0.012	434.95	1467.05	0.049	0.002	0.034	-2.267**
**R2**	119	105	0.997	351	358	17.38	0.009	435.51	1466.50	0.035	0.001	0.043	-2.464**
**R1+R2**	224	194	0.997	551	582	22.99	0.012	435.25	1466.75	0.047	0.002	0.036	-2.431**

- VPg (564 nt)

**Phylogroups**	**N**	**H**	**Hd**	**S**	**η**	**K**	**Π**	**SS**	**NS**	**d_S**	**d_N**	**d_N/d_S**	**Tajima's D[a]**
**Os**	136	94	0.959	151	167	13.42	0.024	116.40	447.60	0.099	0.004	0.043	-1.817*
**Ns**	26	22	0.988	110	112	29.47	0.052	115.63	448.37	0.216	0.010	0.047	0.016ns
**Cs**	14	14	1.000	208	235	63.44	0.112	116.27	447.73	0.424	0.031	0.074	-0.638ns
**Os+Ns+Cs**	176	130	0.975	257	327	37.84	0.067	116.28	447.72	0.267	0.015	0.057	-1.079ns
**R1**	105	66	0.955	140	153	9.65	0.017	116.76	447.24	0.069	0.003	0.051	-2.224**
**R2**	119	73	0.960	145	157	8.07	0.014	116.04	447.96	0.059	0.003	0.042	-2.380**
**R1+R2**	224	137	0.979	182	206	37.04	0.066	116.38	447.62	0.265	0.014	0.052	0.239ns

- NIa (732 nt)

**Phylogroups**	**N**	**H**	**Hd**	**S**	**η**	**K**	**Π**	**SS**	**NS**	**d_S**	**d_N**	**d_N/d_S**	**Tajima's D[a]**
**Os**	136	95	0.956	226	262	22.72	0.031	163.53	568.47	0.106	0.009	0.090	-1.718ns
**Ns**	26	21	0.982	100	106	28.78	0.039	161.48	570.52	0.164	0.004	0.026	0.141ns
**Cs**	14	14	1.000	266	303	83.27	0.114	164.39	567.61	0.422	0.024	0.058	-0.569ns
**Cs**	13	13	1.000	231	257	75.20	0.103	164.40	567.60	0.384	0.021	0.055	-0.422ns
**Os+Ns+Cs**	176	130	0.973	319	420	61.97	0.085	163.30	568.70	0.313	0.019	0.061	-0.492ns
**R1**	105	76	0.975	260	296	18.42	0.025	163.05	568.95	0.091	0.006	0.069	-2.266**
**R2**	119	84	0.987	210	226	9.25	0.013	161.76	570.24	0.044	0.004	0.085	-2.584***
**R1+R2**	224	160	0.991	311	371	75.29	0.102	162.37	569.63	0.390	0.021	0.053	0.682ns

-NIb (1569 nt)

**Phylogroups**	**N**	**H**	**Hd**	**S**	**η**	**K**	**Π**	**SS**	**NS**	**d_S**	**d_N**	**d_N/d_S**	**Tajima's D[a]**
**Os**	136	120	0.995	454	493	44.32	0.028	338.53	1218.47	0.113	0.005	0.044	-1.674ns
**Ns**	26	25	0.997	224	234	57.41	0.037	334.94	1222.06	0.154	0.005	0.030	-0.253ns

**Cs**	14	14	1.000	514	596	159.15	0.102	339.27	1217.73	0.403	0.018	0.046	-0.682ns
**Os+Ns+Cs**	176	159	0.997	657	838	115.14	0.074	338.05	1218.95	0.296	0.012	0.042	-0.684ns
**R1**	105	92	0.995	354	375	20.69	0.013	338.77	1218.23	0.052	0.003	0.050	-2.397**
**R2**	119	110	0.998	342	371	18.98	0.012	335.85	1221.15	0.041	0.004	0.100	-2.419**
**R1+R2**	224	202	0.998	576	656	127.23	0.082	337.22	1219.78	0.322	0.015	0.047	0.513ns

- CP (801 nt)

Phylogroups	N	H	Hd	S	η	K	Π	SS	NS	d_S	d_N	d_N/d_S	Tajima's D[a]
**Os**	136	90	0.953	182	200	18.53	0.023	181.37	619.63	0.070	0.009	0.131	-1.604ns
**Ns**	26	16	0.938	56	57	17.46	0.022	181.66	619.34	0.087	0.003	0.029	0.650ns
**Cs**	14	14	1.000	201	229	57.02	0.071	182.56	618.44	0.217	0.028	0.129	-0.937ns
**Os+Ns+Cs**	176	120	0.971	279	344	41.08	0.051	181.51	619.49	0.169	0.016	0.097	-1.012ns
**R1**	105	76	0.982	154	168	9.28	0.011	182.52	618.48	0.036	0.004	0.114	-2.369**
**R2**	119	89	0.988	191	207	32.07	0.040	171.88	619.12	0.130	0.013	0.010	-0.657ns
**R1+R2**	222	164	0.993	235	265	38.79	0.048	182.18	618.82	0.159	0.016	0.098	-0.369ns

[a]Nd: Not determined; [ns]: Not significant

N: Number of sequences, H: Number of haplotypes, Hd: Haplotype diversity, S: Number of polymorphic (Segregating) sites, η: Number of mutations, K: Average number of nucleotide differences between sequences, π: Nucleotide diversity, SS: Number of synonymous sites, NS: Number of non-synonymous sites, d_N: Non-synonymous nucleotide diversity, d_S: Synonymous nucleotide diversity and d_N/d_S: the ratio of non-synonymous nucleotide diversity to synonymous nucleotide diversity.

Population genetics of TuMV

S2.1 Genetic diversity of TuMV populations based on the polyprotein.

Phylogroups	N	H	Hd	S	η	K	π	SS	NS	d_S	d_N	d_N/d_S	Tajima's D[a]
**WorldB**	89	85	0.998	3729	4503	495.55	0.052	2146.73	7345.27	0.190	0.012	0.064	-1.530ns
**BRs**	44	44	1.000	2812	3214	498.89	0.053	2153.66	7338.35	0.196	0.010	0.053	-1.208ns
**BasalB**	12	12	1.000	3482	4164	1414.68	0.149	2135.15	7347.85	0.543	0.035	0.064	0.123ns
**Iran**	22	22	1.000	2783	3110	1008.93	0.106	2142.21	7349.80	0.382	0.026	0.068	0.753ns
**TuMV n-rec**	167	163	0.999	4667	6976	1250.23	0.132	2145.52	7337.49	0.469	0.033	0.071	0.066ns

**TuMV-recs**	301	301	1.000	5054	7982	1460.20	0.154	2134.91	7324.09	0.551	0.039	0.071	0.469ns

S2.2 Genetic diversity and genetic distances within and between the TuMV populations.

	**Within populations**	**Between populations**			
		**WorldB**	**BRs**	**BasalB**	**Iran**
**WorldB**	0.0522 (0.001)	…	0.002	0.002	0.003
**BRs**	0.0526 (0.001)	0.1608	…	0.002	0.003
**BasalB**	0.1492 (0.002)	0.1922	0.1896	…	0.003
**Iran**	0.1063 (0.002)	0.1910	0.1848	0.1935	…

S2.3 Genetic differentiation measurement for TuMV populations

**Phylogroups**	[a]**Ks*** (***P*-value**)	[a]**Z*** (***P*-value**)	[a]**Snn** (***P*-value**)	[b]**Fst**
WorldB (n= 89) vs. BRs (n= 44)	5.752 (0.000***)	7.425 (0.000***)	1.000 (0.000***)	0.674
WorldB (n= 89) vs. BasalB (n= 12)	6.031 (0.000***)	7.363 (0.000***)	1.000 (0.000***)	0.476
WorldB (n= 89) vs. Iran (n= 22)	6.024 (0.000***)	7.389 (0.000***)	1.000 (0.000***)	0.585
BRs (n= 44) vs. BasalB (n= 12)	5.750 (0.000***)	6.032 (0.000***)	1.000 (0.000***)	0.468
BRs (n= 44) vs. Iran (n= 22)	5.783 (0.000***)	6.179 (0.000***)	1.000 (0.000***)	0.570
BasalB (n= 12) vs. Iran (n= 22)	6.719 (0.000***)	4.744 (0.000***)	1.000 (0.000***)	0.340

[ns], not significant; *, 0.01<P<0.05; **, 0.001<P<0.01; ***, P<0.001; [a]K*, Z*, and Snn are test statistics of genetic differentiation; [b]F_{ST}, coefficient of gene differentiation, which measures inter-population diversity

S2.4 Genetic diversity of TuMV populations based on each gene separately

- P1 (1086 nt)

**Phylogroups**	**N**	**H**	**Hd**	**S**	**η**	**K**	**Π**	**SS**	**NS**	d_S	d_N	d_N/d_S	**Tajima's D**[a]
**WorldB**	89	68	0.987	597	729	72.17	0.066	251.97	834.03	0.171	0.035	0.205	-1.715ns
**BRs**	44	38	0.994	448	518	73.02	0.067	257.38	828.63	0.175	0.034	0.195	-1.432ns
**BasalB**	12	12	1.000	546	674	215.09	0.198	250.78	835.22	0.473	0.116	0.244	-0.171ns
**Iran**	22	21	0.996	404	450	144.76	0.133	254.05	831.95	0.337	0.071	0.211	0.708ns
**TuMV n-rec**	167	139	0.996	779	1169	177.06	0.163	253.58	832.42	0.409	0.088	0.215	-0.450ns

**TuMV-recs**	301	282	0.999	837	1366	203.41	0.187	251.13	834.87	0.470	0.102	0.217	-0.202ns

- HC-Pro (1374 nt)

Phylogroups	N	H	Hd	S	η	K	π	SS	NS	d_S	d_N	d_N/d_S	Tajima's D[a]
**WorldB**	89	71	0.985	516	615	74.96	0.055	312.93	1061.07	0.221	0.006	0.026	-1.316ns
**BRs**	44	38	0.990	459	530	83.92	0.061	312.25	1061.75	0.249	0.006	0.023	-1.152ns
**BasalB**	12	12	1.000	490	582	200.79	0.146	308.03	1065.97	0.584	0.019	0.033	0.197ns
**Iran**	22	20	0.991	381	419	137.79	0.100	309.19	1064.81	0.397	0.014	0.030	0.815ns
**TuMV n-rec**	167	141	0.995	643	982	201.25	0.146	311.90	1062.10	0.557	0.026	0.046	0.543ns
**TuMV-recs**	301	280	0.999	692	1114	234.51	0.171	310.09	1063.91	0.656	0.029	0.044	1.011ns

- P3 (1065 nt)

Phylogroups	N	H	Hd	S	η	K	Π	SS	NS	d_S	d_N	d_N/d_S	Tajima's D[a]
**WorldB**	89	70	0.993	491	627	74.32	0.070	235.10	829.90	0.215	0.029	0.133	-1.374ns
**BRs**	44	34	0.986	363	414	68.61	0.064	238.61	826.39	0.222	0.019	0.085	-1.031ns
**BasalB**	12	12	1.000	429	514	169.54	0.159	238.57	826.43	0.537	0.050	0.093	-0.018ns
**Iran**	22	18	0.983	366	406	136.22	0.128	230.09	825.91	0.392	0.051	0.131	0.915ns
**TuMV n-rec**	167	134	0.997	617	942	176.79	0.166	236.80	828.20	0.498	0.070	0.142	0.223ns
**TuMV-recs**	301	278	0.999	652	1087	202.82	0.190	238.72	826.28	0.574	0.079	0.138	0.539ns

- PIPO (189 nt)

Phylogroups	N	H	Hd	S	η	K	Π	SS	NS	d_S	d_N	d_N/d_S	Tajima's D[a]
**WorldB**	89	46	0.923	56	62	5.68	0.030	39.63	149.37	0.067	0.020	0.302	-1.748ns
**BRs**	44	13	0.719	29	29	4.62	0.024	39.81	149.19	0.068	0.013	0.185	-1.039ns
**BasalB**	12	9	0.939	38	39	14.26	0.075	40.76	148.24	0.248	0.028	0.112	0.473ns
**Iran**	22	14	0.935	31	32	11.21	0.059	41.34	147.66	0.127	0.040	0.316	1.069ns
**TuMV n-rec**	167	82	0.958	84	101	14.64	0.077	39.98	149.02	0.176	0.051	0.289	-0.548ns
**TuMV-recs**	301	172	0.988	85	103	17.97	0.095	40.05	148.95	0.238	0.056	0.237	0.289ns

- CI (1932 nt)

Phylogroups	N	H	Hd	S	η	K	π	SS	NS	d_S	d_N	d_N/d_S	Tajima's D[a]
**WorldB**	89	73	0.991	704	834	95.34	0.049	449.11	1482.89	0.195	0.005	0.027	-1.449ns
**BRs**	44	37	0.990	537	623	93.83	0.049	447.23	1484.77	0.199	0.003	0.017	-1.279ns
**BasalB**	12	12	1.000	658	773	267.68	0.138	447.38	1484.62	0.546	0.016	0.029	0.216ns
**Iran**	22	22	1.000	530	599	192.55	0.099	446.82	1485.18	0.380	0.0152	0.040	0.706ns
**TuMV n-rec**	167	144	0.997	855	1257	229.26	0.119	448.19	1483.81	0.468	0.013	0.028	0.125ns
**TuMV-recs**	301	287	0.999	917	1428	277.69	0.146	440.45	1461.55	0.573	0.017	0.030	0.695ns

- VPg (576 nt)

Phylogroups	N	H	Hd	S	η	K	π	SS	NS	d_S	d_N	d_N/d_S	Tajima's D[a]
**WorldB**	89	69	0.987	241	291	31.77	0.055	126.65	449.35	0.203	0.014	0.067	-1.526ns
**BRs**	44	33	0.985	158	184	30.12	0.052	127.74	448.26	0.182	0.015	0.082	-1.055ns
**BasalB**	12	12	1.000	227	275	96.89	0.168	128.42	447.58	0.614	0.040	0.065	0.301ns
**Iran**	22	20	0.987	190	208	65.35	0.113	129.12	446.88	0.411	0.027	0.065	0.593ns
**TuMV n-rec**	167	134	0.995	294	463	81.85	0.142	127.39	448.61	0.503	0.039	0.078	0.021ns
**TuMV-recs**	301	263	0.998	322	536	96.68	0.168	126.72	449.28	0.580	0.051	0.088	0.415ns

- NIa (729 nt)

Phylogroups	N	H	Hd	S	η	K	π	SS	NS	d_S	d_N	d_N/d_S	Tajima's D[a]
**WorldB**	89	63	0.966	262	310	31.22	0.043	161.61	567.39	0.179	0.004	0.022	-1.673ns
**BRs**	44	30	0.982	204	227	40.09	0.055	161.69	567.31	0.230	0.005	0.022	-0.852ns
**BasalB**	12	11	0.985	263	319	111.88	0.153	161.19	567.81	0.625	0.019	0.031	0.278ns
**Iran**	22	19	0.983	211	244	79.28	0.109	160.58	568.42	0.468	0.007	0.015	0.754ns
**TuMV n-rec**	167	123	0.988	321	472	91.73	0.126	161.47	567.53	0.516	0.015	0.029	0.345ns
**TuMV-recs**	301	257	0.998	343	517	104.96	0.144	161.92	567.08	0.597	0.015	0.025	0.859ns

-NIb (1551 nt)

Phylogroups	N	H	Hd	S	η	K	π	SS	NS	d_S	d_N	d_N/d_S	Tajima's D[a]
**WorldB**	89	68	0.951	562	663	71.12	0.046	347.29	1203.71	0.183	0.006	0.033	-1.570ns
**BRs**	44	40	0.996	392	435	68.19	0.044	349.31	1201.69	0.177	0.005	0.029	-1.176ns
**BasalB**	12	12	1.000	542	631	227.22	0.146	344.75	1206.25	0.585	0.021	0.036	0.413ns
**Iran**	22	19	0.987	431	492	157.72	0.102	348.33	1202.67	0.400	0.015	0.037	0.692ns
**TuMV n-rec**	167	139	0.986	693	1010	181.12	0.117	347.78	1203.22	0.466	0.016	0.034	0.068ns
**TuMV-recs**	301	277	0.999	763	1155	210.26	0.135	346.73	1204.27	0.542	0.018	0.034	0.450ns

- CP (864 nt)

Phylogroups	N	H	Hd	S	η	K	Π	SS	NS	d_S	d_N	d_N/d_S	Tajima's D[a]
**WorldB**	89	66	0.970	233	274	26.63	0.031	189.89	674.11	0.119	0.006	0.049	-1.731ns
**BRs**	44	30	0.970	146	157	2.71	0.026	189.98	674.02	0.105	0.004	0.038	-1.354ns
**BasalB**	12	11	0.985	209	244	75.12	0.088	184.57	670.43	0.338	0.019	0.056	-0.330ns
**Iran**	22	19	0.974	169	180	57.85	0.067	185.36	678.64	0.251	0.017	0.066	0.699ns
**TuMV n-rec**	167	125	0.988	317	428	66.69	0.078	187.33	667.67	0.297	0.016	0.055	-0.367ns
**TuMV-recs**	301	264	0.998	362	508	75.74	0.088	187.71	673.29	0.339	0.018	0.053	-0.197ns

[a]Nd: Not determined; ns: Not significant

N: Number of sequences, H: Number of haplotypes, Hd: Haplotype diversity, S: Number of polymorphic (Segregating) sites, η: Number of mutations, K: Average number of nucleotide differences between sequences, π: Nucleotide diversity, SS: Number of synonymous sites, NS: Number of non-synonymous sites, d_N: Non-synonymous nucleotide diversity, d_S: Synonymous nucleotide diversity and d_N/d_S: the ratio of non-synonymous nucleotide diversity to synonymous nucleotide diversity.

Population genetics of Sweet potato viruses

S3.1 Genetic diversity of Sweet potato viruses population based on the polyprotein

Phylogroups	N	H	Hd	S	η	K	π	SS	NS	d_S	d_N	d_N/d_S	Tajima's D[a]
**SPVG**	10	10	1.000	2525	2641	566.20	0.053	2330.13	8097.87	0.176	0.019	0.109	-1.976**
**SPFMV**	24	24	1.000	2116	2235	351.63	0.033	2371.90	8062.10	0.115	0.010	0.086	-1.674ns
**SPLV**	3	3	1.000	823	830	551.00	0.057	2205.56	7535.44	0.210	0.011	0.055	ND

**SPV2**	10	10	1.000	894	921	214.00	0.020	2308.27	8089.73	0.063	0.008	0.134	-1.719ns
**SPVC**	12	12	1.000	1592	1647	409.92	0.039	2370.25	8072.75	0.132	0.012	0.090	-1.175ns
**Total n-rec**	59	59	1.000	6277	10487	2495.12	0.258	2168.09	7476.91	0.606	0.158	0.260	0.379ns
**SPV-recs**	50	48	0.998	5972	9794	1916.05	0.205	2111.99	7233.01	0.537	0.108	0.201	-0.453ns

[a]Nd: Not determined; ns: Not significant

N: Number of sequences, H: Number of haplotypes, Hd: Haplotype diversity, S: Number of polymorphic (Segregating) sites, η: Number of mutations, K: Average number of nucleotide differences between sequences, π: Nucleotide diversity, SS: Number of synonymous sites, NS: Number of non-synonymous sites, d_N: Non-synonymous nucleotide diversity, d_S: Synonymous nucleotide diversity

S3.2 Genetic diversity and genetic distance values within and between the Sweet potato viruses

	**Within populations**	**Between populations**				
		**SPFMV**	**SPVC**	**SPVG**	**SPV2**	**SPLV**
**SPFMV**	0.033 (0.001)	…	0.004	0.004	0.004	0.005
**SPVC**	0.039 (0.001)	0.269	…	0.003	0.004	0.004
**SPVG**	0.053 (0.001)	0.342	0.348	…	0.003	0.004
**SPV2**	0.020 (0.001)	0.337	0.346	0.297	…	0.005
**SPLV**	0.057 (0.001)	0.442	0.445	0.435	0.437	…

S3.3 Genetic differentiation measurement for Sweet potato viruses

**Phylogroups**	**[a]Ks* (*P*-value)**	**[a]Z* (*P*-value)**	**[a]Snn (*P*-value)**	**[b]Fst**
SPFMV (n= 24) vs. SPVC (n= 12)	5.548 (0.000***)	4.867 (0.000***)	1.000 (0.000***)	0.948
SPFMV (n= 24) vs. SPVG (n= 10)	5.465 (0.000***)	4.704 (0.000***)	1.000 (0.000***)	0.876
SPFMV (n= 24) vs. SPV2 (n= 10)	5.428 (0.000***)	4.814 (0.000***)	1.000 (0.000***)	0.923
SPFMV (n= 24) vs. SPLV (n= 3)	5.414 (0.000***)	4.655 (0.000***)	1.000 (0.000***)	0.896
SPVC (n= 12) vs. SPVG (n= 10)	5.602 (0.000***)	3.715 (0.000***)	1.000 (0.000***)	0.934
SPVC (n= 12) vs. SPV2 (n= 10)	5.518 (0.000***)	3.740 (0.000***)	1.000 (0.000***)	0.959
SPVC (n= 12) vs. SPLV (n= 3)	5.664 (0.000***)	3.294 (0.000***)	1.000 (0.000***)	0.935

SPVG (n= 10) vs. SPV2 (n= 10)	5.421 (0.000***)	3.535 (0.000***)	1.000 (0.000***)	0.876
SPVG (n= 10) vs. SPLV (n= 3)	5.454 (0.000***)	2.966 (0.000***)	1.000 (0.000***)	0.872
SPV2 (n= 10) vs. SPLV (n= 3)	5.312 (0.000***)	2.945 (0.000***)	1.000 (0.000***)	0.912

[ns], not significant; *, 0.01<P<0.05; **, 0.001<P<0.01; ***, P<0.001; [a]K*, Z*, and Snn are test statistics of genetic differentiation; [b]F_{ST}, coefficient of gene differentiation, which measures inter-population diversity

S3.4 Genetic diversity of Sweet potato viruses population based on the each gene separately

- P1

Virus	N	H	Hd	S	η	K	Π	SS	NS	d_S	d_N	d_N/d_S	Tajima's D[a]
**SPVG**	10	10	1.000	625	655	140.00	0.756	398.37	1455.63	0.179	0.047	0.262	-1.977**
**SPFMV**	24	24	1.000	517	554	85.37	0.043	463.74	1516.26	0.102	0.025	0.244	-1.717ns
**SPVC**	12	12	1.000	374	388	92.22	0.047	450.99	1511.01	0.111	0.028	0.252	-1.331ns
**SPV2**	10	10	1.000	205	209	48.53	0.026	400.48	1447.52	0.069	0.014	0.207	-1.712ns
**SPLV**	3	3	1.000	135	135	90.00	0.068	298.94	1027.06	0.178	0.035	0.200	ND
**Total**	59	59	1.000	1093	1858	413.68	0.317	291.78	1010.22	0.559	0.248	0.443	0.124ns
**SPV-rec**	50	48	0.998	1041	1832	328.04	0.259	294.24	968.76	0.467	0.197	0.421	-0.724ns

- PISPO

Virus	N	H	Hd	S	η	K	Π	SS	NS	d_S	d_N	d_N/d_S	Tajima's D[a]
**SPVG**	10	10	1.000	251	260	54.93	0.080	141.83	542.17	0.063	0.085	1.336	-2.010**
**SPFMV**	24	23	0.996	162	174	28.15	0.041	150.08	539.92	0.036	0.042	1.162	-1.586ns
**SPVC**	12	12	1.000	101	104	24.82	0.036	152.29	537.71	0.039	0.035	0.909	-1.303ns
**SPV2**	10	10	1.000	69	69	16.00	0.023	149.32	534.68	0.025	0.023	0.909	-1.693ns
**SPLV***	-	-	-	-	-	-	-	-	-	-	-	-	-
**Total**	56	55	0.999	459	738	195.11	0.291	144.15	524.85	0.233	0.308	1.322	0.773ns
**SPV-rec**	48	44	0.996	461	721	147.07	0.219	146.17	522.83	0.184	0.229	1.243	-0.347ns

* PISPO was not identified in this virus.

- HC-Pro

Virus	N	H	Hd	S	η	K	Π	SS	NS	d_S	d_N	d_N/d_S	Tajima's D[a]

**SPVG**	10	10	1.000	376	398	84.68	0.062	304.02	1063.98	0.192	0.025	0.131	-1.993**
**SPFMV**	24	23	0.996	317	343	56.48	0.041	298.36	1075.64	0.159	0.008	0.052	-1.553ns
**SPVC**	12	12	1.000	193	201	50.68	0.037	289.81	1084.19	0.137	0.010	0.074	-1.121ns
**SPV2**	10	10	1.000	120	121	29.56	0.021	293.92	1080.08	0.070	0.008	0.116	-1.534ns
**SPLV**	3	3	1.000	94	95	63.00	0.046	304.00	1070.00	0.192	0.004	0.022	ND
**Total**	59	58	0.999	847	1454	343.44	0.251	296.01	1071.99	0.651	0.141	0.216	0.350ns
**SPV-rec**	50	47	0.998	838	1369	274.72	0.199	295.01	1078.99	0.587	0.094	0.160	-0.370ns

- P3

**Virus**	**N**	**H**	**Hd**	**S**	**η**	**K**	**Π**	**SS**	**NS**	d_S	d_N	d_N/d_S	**Tajima's D**[a]
**SPVG**	10	10	1.000	250	258	55.00	0.053	221.90	804.10	0.178	0.019	0.106	-1.983**
**SPFMV**	24	23	0.996	155	157	22.60	0.021	245.68	810.32	0.074	0.005	0.073	-1.850ns
**SPVC**	12	12	1.000	154	157	38.92	0.037	246.17	809.83	0.121	0.011	0.092	-1.178ns
**SPV2**	10	10	1.000	78	79	18.35	0.017	228.63	827.37	0.052	0.008	0.146	-1.692ns
**SPLV**	3	3	1.000	152	153	101.66	0.096	243.83	812.17	0.339	0.023	0.068	ND
**Total**	59	58	0.999	821	1375	329.96	0.321	231.77	794.23	0.577	0.246	0.427	0.413ns
**SPV-rec**	50	43	0.993	790	1286	234.29	0.232	234.43	773.57	0.484	0.156	0.322	-0.673ns

- PIPO

**Virus**	**N**	**H**	**Hd**	**S**	**η**	**K**	**Π**	**SS**	**NS**	d_S	d_N	d_N/d_S	**Tajima's D**[a]
**SPVG**	10	6	0.778	44	45	9.44	0.050	40.33	148.67	0.126	0.029	0.235	-1.979*
**SPFMV**	24	8	0.562	8	8	1.116	0.005	41.21	156.79	0.025	0.001	0.021	-1.537ns
**SPVC**	12	11	0.985	23	24	5.576	0.028	46.60	151.40	0.023	0.0287	1.253	-1.326ns
**SPV2**	10	8	0.933	13	13	2.87	0.015	42.47	146.53	0.034	0.009	0.277	-1.712ns
**SPLV**	3	3	1.000	22	22	14.66	0.073	49.06	151.94	0.095	0.065	0.689	0.531ns
**Total**	59	34	0.911	150	229	56.66	0.320	40.25	136.75	0.445	0.283	0.634	0.531ns
**SPV-rec**	50	50	26	0.955	119	176	32.42	0.210	34.20	118.80	0.282	0.190	0.673

- CI

**Virus**	**N**	**H**	**Hd**	**S**	**η**	**K**	**Π**	**SS**	**NS**	d_S	d_N	d_N/d_S	**Tajima's D**[a]
**SPVG**	10	10	1.000	428	452	95.82	0.049	442.98	1486.02	0.186	0.009	0.049	-2.005**

**SPFMV**	24	24	1.000	344	357	57.81	0.030	447.70	1472.30	0.115	0.004	0.035	-1.595ns
**SPVC**	12	12	1.000	279	290	74.77	0.039	451.08	1477.92	0.150	0.005	0.031	-1.043ns
**SPV2**	10	10	1.000	114	119	26.73	0.014	435.70	1493.30	0.053	0.002	0.047	-1.809*
**SPLV**	3	3	1.000	171	173	114.66	0.059	450.50	1493.50	0.238	0.005	0.020	ND
**Total**	59	59	1.000	1124	1815	451.83	0.234	444.38	1475.62	0.619	0.119	0.192	0.563ns
**SPV-rec**	50	46	0.997	1115	1748	359.83	359.83	447.85	1487.15	0.534	0.080	0.151	-0.285ns

- VPg

**Virus**	**N**	**H**	**Hd**	**S**	**η**	**K**	**Π**	**SS**	**NS**	d_S	d_N	d_N/d_S	**Tajima's D[a]**
**SPVG**	10	10	1.000	131	135	29.48	0.051	133.18	442.82	0.184	0.012	0.063	-1.899*
**SPFMV**	24	22	0.993	130	133	21.90	0.038	127.51	439.49	0.148	0.007	0.045	-1.536ns
**SPVC**	12	12	1.000	93	95	22.77	0.039	131.86	444.14	0.140	0.009	0.069	-1.285ns
**SPV2**	10	10	1.000	56	56	13.82	0.024	129.50	452.50	0.084	0.010	0.078	-1.479ns
**SPLV**	3	3	1.000	31	31	20.67	0.036	130.06	448.94	0.149	0.003	0.020	ND
**Total**	59	57	0.999	364	592	145.15	0.253	127.96	439.04	0.629	0.145	0.230	0.498ns
**SPV-rec**	50	47	0.998	348	560	115.27	0.202	130.10	439.90	0.564	0.095	0.168	-0.284ns

- NIa

**Virus**	**N**	**H**	**Hd**	**S**	**η**	**K**	**Π**	**SS**	**NS**	d_S	d_N	d_N/d_S	**Tajima's D[a]**
**SPVG**	10	10	1.000	143	147	30.82	0.042	154.48	574	0.183	0.004	0.023	-2.024**
**SPFMV**	24	22	0.993	156	165	25.87	0.036	159.85	560.15	0.148	0.004	0.025	-1.660ns
**SPVC**	12	12	1.000	104	105	27.32	0.037	163.15	565.85	0.154	0.004	0.024	-0.999ns
**SPV2**	10	10	1.000	47	48	11.42	0.016	160.40	568.60	0.064	0.002	0.032	-1.595ns
**SPLV**	3	3	1.000	41	41	27.33	0.037	159.94	569.06	0.160	0.003	0.005	ND
**Total**	59	57	0.999	417	677	167.84	0.231	158.16	561.84	0.657	0.109	0.167	0.544ns
**SPV-rec**	50	45	0.995	411	664	134.38	0.184	161.19	567.81	0.591	0.069	0.116	-0.341ns

-NIb

**Virus**	**N**	**H**	**Hd**	**S**	**η**	**K**	**Π**	**SS**	**NS**	d_S	d_N	d_N/d_S	**Tajima's D[a]**
**SPVG**	10	10	1.000	323	333	71.71	0.046	351.72	1211.28	0.171	0.010	0.057	-1.955*

**SPFMV**	24	24	1.000	292	304	48.29	0.031	345.60	1217.40	0.118	0.006	0.052	-1.639ns
**SPVC**	12	12	1.000	242	252	63.50	0.041	348.61	1214.39	0.155	0.008	0.049	-1.124ns
**SPV2**	10	10	1.000	104	109	24.38	0.016	350.17	1212.83	0.058	0.003	0.057	-1.822*
**SPLV**	3	3	1.000	93	94	62.33	0.040	346.00	1208.00	0.167	0.004	0.023	ND
**Total**	59	59	1.000	884	1491	367.70	0.237	345.29	1202.71	0.618	0.127	0.205	0.523ns
**SPV-rec**	50	48	0.998	895	1472	299.77	0.193	343.33	1210.67	0.567	0.086	0.152	-0.321ns

- CP

**Virus**	**N**	**H**	**Hd**	**S**	**η**	**K**	**Π**	**SS**	**NS**	**d_S**	**d_N**	**d_N/d_S**	**Tajima's D[a]**
**SPVG**	10	10	1.000	183	190	42.98	0.040	255.58	809.42	0.128	0.013	0.097	-1.795*
**SPFMV**	24	22	0.993	150	164	25.63	0.027	216.01	725.99	0.084	0.010	0.123	-1.666ns
**SPVC**	12	12	1.000	112	117	27.33	0.029	217.08	721.92	0.092	0.010	0.111	-1.376ns
**SPV2**	10	10	1.000	100	105	25.11	0.025	233.80	762.20	0.052	0.017	0.323	-1.603ns
**SPLV**	3	3	1.000	69	70	46.33	0.053	206.67	672.33	0.182	0.013	0.070	ND
**Total**	59	57	0.999	531	832	184.94	0.212	200.12	672.88	0.530	0.117	0.220	0.117ns
**SPV-rec**	50	46	0.997	396	647	119.40	0.180	151.59	511.41	0.492	0.087	0.177	-0.632ns

[a]Nd: Not determined; [ns]: Not significant

N: Number of sequences, H: Number of haplotypes, Hd: Haplotype diversity, S: Number of polymorphic (Segregating) sites, η: Number of mutations, K: Average number of nucleotide differences between sequences, π: Nucleotide diversity, SS: Number of synonymous sites, NS: Number of non-synonymous sites, d_N: Non-synonymous nucleotide diversity, d_S: Synonymous nucleotide diversity and d_N/d_S: the ratio of non-synonymous nucleotide diversity to synonymous nucleotide diversity, ND: Not determined.

Population genetics of HarMV

S4.1 Genetic diversity of HarMV population based on the polyprotein and each gene separately

**Proteins**	**N**	**H**	**Hd**	**S**	**η**	**K**	**π**	**SS**	**NS**	**d_S**	**d_N**	**d_N/d_S**	**Tajima's D[a]**
**Polyprotein**	10	9	0.978	2952	3578	1164.20	0.139	1843.13	6553.87	0.494	0.039	0.078	-0.399 ns
**P1**	10	8	0.956	247	302	93.556	0.181	116.33	399.67	0.488	0.092	0.188	-0.618 ns
**HC-Pro**	10	8	0.956	475	580	185.80	0.135	297.88	1073.12	0.523	0.028	0.054	-0.469 ns
**P3**	10	8	0.956	392	459	150.04	0.143	228.87	818.13	0.436	0.062	0.141	-0.377 ns
**PIPO**	10	8	0.956	60	67	21.42	0.096	48.58	173.42	0.150	0.082	0.544	-0.469 ns
**CI**	10	9	0.978	603	739	245.26	0.129	432.17	1469.83	0.502	0.019	0.038	-0.306 ns

**VPg**	10	8	0.956	204	241	77.22	0.135	125.92	444.08	0.480	0.038	0.079	-0.467 ns
**NIa**	10	9	0.978	250	306	97.93	0.134	157.45	571.55	0.493	0.035	0.072	-0.473 ns
**NIb**	10	9	0.978	547	667	221.07	0.143	334.13	1216.87	0.514	0.041	0.079	-0.313 ns
**CP**	10	8	0.956	123	148	48.15	0.122	80.52	315.48	0.433	0.042	0.097	-0.395 ns

[a]ns: Not significant

N: Number of sequences, H: Number of haplotypes, Hd: Haplotype diversity, S: Number of polymorphic (Segregating) sites, η: Number of mutations, K: Average number of nucleotide differences between sequences, π: Nucleotide diversity, SS: Number of synonymous sites, NS: Number of non-synonymous sites, d_N: Non-synonymous nucleotide diversity, d_S: Synonymous nucleotide diversity
